# Pancreatitis Unveiled: Linking Pathogenesis, Organ Failure, and Emerging Predictive Biomarkers

**DOI:** 10.7759/cureus.98838

**Published:** 2025-12-09

**Authors:** Prasanna Kumar Anbazhagan, Hari Prasad Naidu Boyapati, Sidra Tahreem Hashmi Syeda, Arivudainambi Saravanan, Divya Payidiparty

**Affiliations:** 1 General Surgery, Ashford and St Peter's Hospitals NHS Foundation Trust, Chertsey, GBR; 2 Internal Medicine, PES Institute of Medical Sciences and Research, Kuppam, IND; 3 Family Medicine, Jackson Park Hospital, Chicago, USA; 4 Family Medicine, Deccan College of Medical Sciences, Hyderabad, IND; 5 Internal Medicine, Stanley Medical College, Chennai, IND; 6 Research, SDM College of Medical Sciences and Hospital, Dharwad, IND

**Keywords:** acute pancreatitis, organ failure, predictive biomarkers, serum biomarkers, systemic inflammatory response syndrome

## Abstract

Acute pancreatitis is an inflammatory condition that starts as a mild illness but can progress to become fatal through multi-organ failure with high death rates. The APACHE II and BISAP scoring systems help identify patient risks, but their ability to forecast early deterioration remains limited during the initial hours following admission when critical decisions need to be made. This review examines the mechanisms linking pancreatic injury to systemic inflammation and organ failure, outlining the distinction between early sterile organ failure and later sepsis-related failure. Clinical patterns of organ dysfunction correlate strongly with the inflammatory response, and early indicators such as systemic inflammatory response syndrome duration, rising blood urea nitrogen, hemoconcentration, and increasing CRP provide useful predictors of disease severity. Emerging biomarkers, including IL-6, IL-8, circulating histones, angiopoietin-2, arterial lactate, and albumin-based ratios, show promise for forecasting persistent organ failure. Integrating these markers with machine learning algorithms and multi-cytokine panels enhances predictive accuracy for patient risk assessment and early intensive care unit admission. Although promising, most of these biomarkers and AI-based tools remain investigational and require further multicenter validation. Future research should prioritize standardized assay methods and prospective evaluation of combined clinical-biomarker-AI models to determine their true impact on early diagnosis, triage, and patient outcomes.

## Introduction and background

Pancreatitis is characterized by inflammation of the pancreatic parenchyma and can manifest as either acute pancreatitis (AP) or chronic pancreatitis (CP). AP is a medical emergency that poses a significant risk to the patient’s life, whereas CP involves progressive fibrosis of pancreatic parenchyma, leading to long-term functional impairment. Pancreatitis represents one of the most common causes of hospitalizations related to gastrointestinal diseases, associated with a significant amount of morbidity, mortality, and economic burden [1]. It can affect individuals of any age, and if not managed properly, it can result in a huge financial burden on patients, especially in low- and middle-income countries [2]. Globally, the incidence of AP is estimated at 33.74 per 100,000 person-years (95% CI 23.33-48.81) [3]. The mortality rate ranges from 3% in cases of mild edematous pancreatitis to 20% in pancreatic necrosis [4].

The clinical spectrum of pancreatitis can range from a single episode of AP to recurrent attacks, particularly in the presence of risk factors such as consuming alcohol on a regular basis or having a genetic predisposition, which can ultimately result in CP [5]. The diagnosis of AP is based on sudden onset of severe abdominal pain radiating to the back, elevated serum amylase and/or lipase levels at least three times above the upper limit of normal, and characteristic findings on ultrasound or MRI [6].

AP can lead to a range of complications, both local and systemic. Local complications include early events such as peripancreatic fluid collection and pancreatic or peripancreatic necrosis, with about one-third of these necrotic areas becoming infected [7]. In approximately 20% of cases, systemic complications arise, manifesting as organ failure, which indicates severe AP (SAP). This organ failure can develop early in the disease course, but it may also occur later due to sepsis resulting from infected pancreatic necrosis (IPN) [8]. Sepsis, driven by a dysregulated host response to infection, leads to organ malfunction and remains the leading cause of ICU deaths worldwide. Predicting organ failure in AP remains a significant clinical challenge. Current scoring systems often delay the detection of severe cases and subsequent treatment due to their late applicability and limited sensitivity.

The central challenge in AP is the progression from local pancreatic injury to systemic inflammation and organ failure, which drives morbidity, mortality, and the clinical need for early prediction. In this article, we critically evaluate the underlying pathophysiological mechanisms that connect AP to organ failure while also assessing emerging research on novel biomarkers with the potential to predict organ failure early. This review highlights the growing importance of novel biomarkers in organ failure prediction and AI-based models in improving early identification and management of SAP.

To ensure a comprehensive and evidence-based discussion, we conducted a structured literature search of PubMed-indexed journals to identify studies published between January 2001 and the present. The following keywords and Medical Subject Headings (MeSH) terms were used in various combinations: “acute pancreatitis”, “organ failure”, “emerging predictive biomarkers”, “machine learning”, “artificial intelligence”, “predictive models”, and “systemic inflammatory response syndrome”. Only English-language articles involving human subjects were included, with priority given to original research, systematic reviews, meta-analyses, and clinical guidelines relevant to AP. Studies with uncertain methodological details or unclear outcomes were excluded. Titles and abstracts were screened manually, followed by full-text review of eligible articles. Data extracted included study design, patient population, biomarkers or predictive models evaluated, and key outcomes. The methodological quality and risk of bias were qualitatively assessed, focusing on patient selection, outcome definitions, analytical approach, and validation methods. Because this review was conducted as a narrative synthesis, no quantitative pooling, meta-analysis, or meta-regression of data was performed. Reported numerical values (e.g., areas under the curve (AUCs), sensitivity, specificity, and mortality rates) are cited directly from the respective primary studies and were not reanalyzed.

## Review

Pathogenesis and inflammatory response in AP

The pathogenesis of pancreatitis involves several mechanisms of injury. Premature activation of pancreatic proenzymes, such as trypsinogen to trypsin, leads to pancreatic tissue autodigestion and pancreatitis. The failure of intracellular protection systems to prevent activation of trypsinogen and limit trypsin activity leads to AP [9]. Normally, trypsin activity is inhibited by the serine protease inhibitor Kazal type 1 (SPINK1) and further regulated by breakdown through chymotrypsin C (CTRC) and cathepsin L. However, a failure in this protective mechanism can lead to pancreatitis. Additionally, autoactivation or activation by lysosomal cysteine protease cathepsin B can trigger premature intra-pancreatic trypsinogen activation, further contributing to pancreatitis [10-12]. A multitude of genes are responsible for recurrent pancreatitis. For example, gain-of-function mutations in the cystic fibrosis transmembrane conductance regulator (CFTR) gene and cationic trypsinogen gene (PRSS1) are commonly associated with the development of pancreatitis.

**Figure 1:**
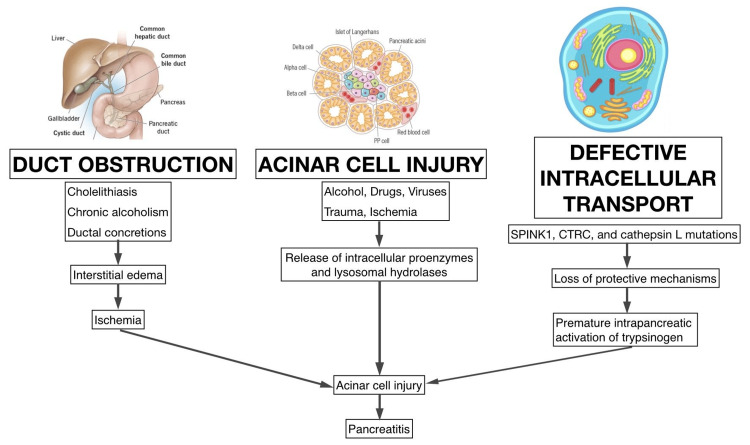
Pathophysiological pathways leading to AP AP, acute pancreatitis; CTRC, chymotrypsin C; SPINK1, serine protease inhibitor Kazal type 1 Image credit: Prasanna Kumar Anbazhagan

Acinar cell injury, resulting from toxins, ischemia, and infections, leads to the release of intracellular calcium, which in turn activates digestive enzymes, causing pancreatitis. Ductal obstruction caused by gallstones or strictures increases pancreatic ductal pressure, causing bile reflux and disrupting the normal enzyme production, thereby causing cellular damage and inflammation. This is due to the co-localization of digestive and lysosomal enzymes intracellularly [13]. The pathogenesis is further explained in Figure 1.

Systemic inflammatory cascade

Local to Systemic Transition

The inflammation in pancreatitis occurs in two stages. First, during the intra-acinar stage, damage and inflammation occur within the acinar cells. Following this, in the extra-acinar stage, the inflammation spreads beyond the acinar cells and spills to local tissue, spreading to the systemic level [14,15]. This escalation leads to increased morbidity and potentially death. This is due to the proinflammatory cytokines produced by the pancreatic acinar cells [16], likely in response to digestive enzymes in the pancreatic parenchyma. Proinflammatory cytokines, such as TNF-α and IL-1β, are released from the peritoneal cavity into circulation via the portal vein [17,18]. This triggers two overlapping systemic responses: the systemic inflammatory response syndrome (SIRS) and the compensatory anti-inflammatory response syndrome (CARS).

SIRS and CARS: Dual Phases of Immune Dysregulation

SIRS causes significant systemic inflammation, multi-organ failure, shock, and early death [19]. Proinflammatory cytokines stimulate the body's vascular endothelium, which increases capillary vein leakage and leukocyte migration into tissues. As adhesion molecules (e.g., CD11b) rise, circulating neutrophils and monocytes release proteolytic enzymes and oxygen radicals that harm vascular endothelium and organ parenchymal cells [20]. Due to increased tissue fluid and reduced microcirculation, essential organs lose oxygen, causing organ dysfunction and failure [21,22].

**Figure 2:**
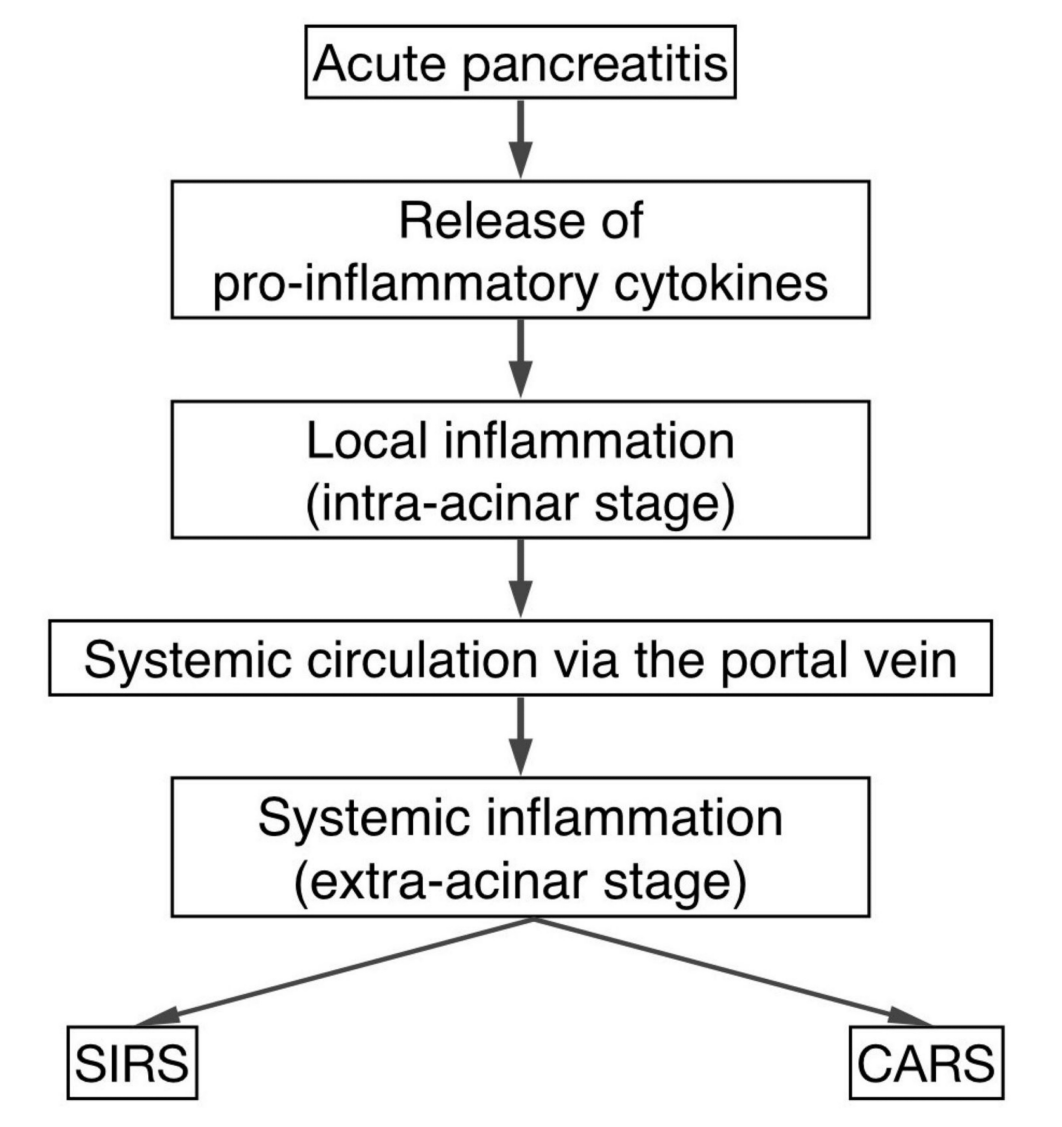
Inflammatory cascade leading to SIRS and CARS in AP AP, acute pancreatitis; CARS, compensatory anti-inflammatory response syndrome; SIRS, systemic inflammatory response syndrome Image credit: Prasanna Kumar Anbazhagan

Simultaneously, the body mounts a CARS phase, which promotes a regulatory anti-inflammatory response, making patients vulnerable to infections [19]. According to Sendler et al. [23], a novel paradigm suggests that both SIRS and CARS initiate early and progress simultaneously rather than sequentially. In support of this theory, they found that SIRS and CARS occurred simultaneously in rats with severe pancreatitis. This conceptual change could help us better comprehend the inflammation in AP [23]. This process is illustrated in Figure 2.

Organ failure in AP

Primary and Secondary Organ Failure

Approximately 80% of pancreatitis patients experience self-resolution, while 20% of patients progress to severe illness with systemic organ failure and pancreatic necrosis, increasing the risk of mortality [24]. AP has an early phase occurring within 14 days after the onset of symptoms. During this early phase, an intense SIRS-driven inflammatory response can lead to multi-organ dysfunction and subsequent organ failure. This organ failure is caused by increased mediator release associated with SIRS, rather than the pancreatic infection [25-28]. This kind of organ failure that happens within a few days of the start of AP is due to sterile inflammation, it carries a high mortality rate [29,30], and is referred to as primary organ failure.

In the later stage, multi-organ failure occurs as a result of sepsis from IPN [31]. This infection risk is heightened because the CARS phase involves an anti-inflammatory response and immunosuppression, making patients more prone to infection, which can lead to IPN [32-35]. During this phase, bacteria translocate from the gastrointestinal tract to the pancreas through hematogenous, lymphatic, and transcoelomic transmission pathways, resulting in IPN [36,37]. IPN carries a mortality rate of about 24% [38,39], and when sepsis from this infection triggers multi-organ dysfunction, it is referred to as secondary organ failure.

Clinical Implications

**Figure 3:**
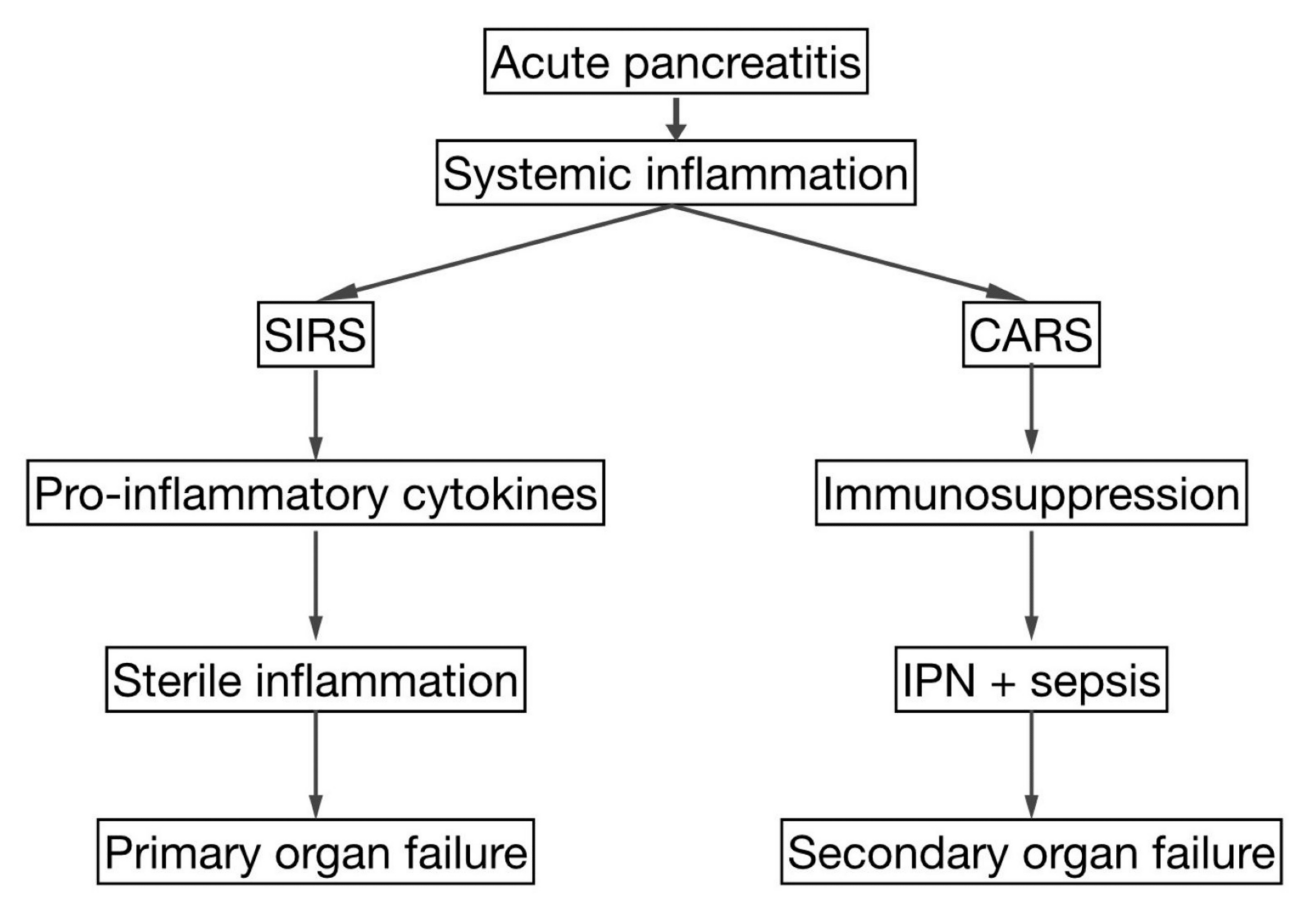
Biphasic progression of systemic inflammation in AP: SIRS and CARS leading to primary and secondary organ failure AP, acute pancreatitis; CARS, compensatory anti-inflammatory response syndrome; IPN, infected pancreatic necrosis; SIRS, systemic inflammatory response syndrome Image credit: Prasanna Kumar Anbazhagan

Primary organ failure leads to early death, whereas secondary organ failure from sepsis has a broader window for timely intervention. The treatment for primary organ failure is supportive, whereas secondary organ failure focuses on sepsis management and surgical intervention. Thus, secondary organ failure has a better prognosis than the primary [8]. This biphasic model of systemic inflammation in AP is illustrated in Figure 3.

Determinants of clinical course and prognosis

Clinical Endpoints and Severity Definitions

The outcome of AP depends most heavily on the development of organ failure and its duration. In 2004, the concepts of transient and persistent organ failure (POF) were explained [40]. POF is defined as a severity of grade ≥2 lasting for more than 48 hours, whereas organ failure lasting less than 48 hours is termed transient organ failure [8].

Consequently, it takes at least three days to diagnose persistent OF, even if a patient develops organ dysfunction within a day of symptom onset. The revised Atlanta categorization of AP severity categorizes SAP as having POF and moderate AP as having transient organ failure [41]. Most commonly affected systems in pancreatitis are the respiratory, renal, and cardiovascular systems [41]. Among these, the most prevalent is respiratory failure [42].

These definitions are critical because they form the basis for triage, monitoring, and therapeutic escalation during the early phase of illness.

Established Prognostic Indicators

Early bedside and laboratory parameters remain critical in anticipating the course of AP and guiding triage decisions.

The duration of SIRS from 24 to 48 hours determines pancreatic necrosis risk and organ failure development and death rates, while time-dependent SIRS progression proves more valuable than isolated measurements [43]. Blood urea nitrogen (BUN) changes during the first 24 hours serve as a basic yet dependable indicator of disease severity because rising BUN values predict fatal outcomes and extended organ system breakdown, thus requiring intensive fluid management [44].

Other laboratory parameters have complementary prognostic value. The presence of hemoconcentration during admission with hematocrit values above 44-45% or nondecreasing values at 24 hours indicates insufficient intravascular fluid replacement and correlates with pancreatic necrosis and organ failure, although findings across cohorts are mixed [45]. CRP levels exceeding 150 mg/L at 48 hours after admission show a strong correlation with pancreatic necrosis and organ failure, although results vary between studies [46]. The BISAP score includes pleural effusion as an imaging finding, which shows an association with poor outcomes but fails to function as an independent risk factor [47].

Collectively, these markers help in early prognostication when used with clinical assessment and validated scoring systems to guide timely interventions for patient-specific care. The combination of patient characteristics, including age and comorbid conditions, together with disease severity indicators (such as necrosis extent and ICU requirements), determines both individual risk and resource requirements. Current guidelines and cohort studies support a risk assessment system that integrates SIRS/BUN trend analysis with modified Marshall and Atlanta severity definitions to determine triage and monitoring needs and intervention levels [48].

Despite their usefulness, established prognostic tools such as BISAP, APACHE II, SIRS duration, BUN trends, hematocrit, and CRP have major limitations during the first 24 hours of AP. Many achieve peak accuracy only after 24-48 hours, limiting their reliability for very early triage. These scores rely on fixed clinical variables and do not capture the rapid immunologic and microcirculatory changes that precede organ dysfunction. CRP rises late, hematocrit depends on fluid status, and BUN reflects systemic hypoperfusion rather than pancreatic injury. These limitations have driven interest in novel biomarkers and AI-based tools, which aim to detect abnormalities earlier. However, they are complementary, not replacements, as most remain in early validation and are not yet ready for routine clinical use.

Severe Complications and Their Clinical Impact

IPN is the second major determinant of poor outcomes. The presence of IPN alongside organ failure results in higher mortality rates than sterile necrosis alone.

If inadequately treated, IPN is associated with markedly increased mortality; however, structured step-up management using percutaneous, endoscopic, and delayed surgical interventions can substantially reduce morbidity and mortality, although risks remain elevated [42]. Recent meta-analyses have confirmed that the step-up approach combining minimally invasive drainage and delayed necrosectomy reduces morbidity and mortality compared with open necrosectomy alone [49].

Thus, defining organ failure early and promptly identifying infected necrosis are the two cornerstones of prognosis and management in SAP.

Promising biomarkers for predicting organ failure in AP

Various studies have explored different biomarkers and clinical indicators that could aid in the early diagnosis of organ failure and SAP. Recent research has identified several biochemical and cellular markers with potential prognostic value, as outlined below.

Among cytokines, it has been found that IL-6 and IL-8 can be early markers in predicting organ failure [50]. In addition, Liu et al. conducted an observational study on 236 patients, finding that early death of immune cells in AP leads to raised histone levels. The study concluded that circulating histones in plasma were a stronger indicator of disease severity and a better predictor of POF and mortality than BISAP [51].

Beyond cytokines and circulating histones, certain clinical and hematologic markers have also shown prognostic value. Brown A et al. found that patients without hemoconcentration seldom had pancreatic necrosis or organ failure [45]. In a retrospective study of 158 patients with AP, Li et al. demonstrated that serum albumin upon admission independently predicted POF, providing a simple and rapid tool for early risk stratification [52].

Furthermore, Shu et al. retrospectively evaluated data from 329 patients and found that SAP patients with increased arterial lactate levels had higher morbidity and mortality rates. Serial lactate monitoring might also reveal the progression of the disease. This study suggests that arterial lactate monitoring may be a simple, fast technique for identifying high-risk SAP patients [53].

A prospective study by Zhang et al. evaluated 120 AP patients and found that high serum angiopoietin-2 levels on admission could signify POF in AP [54]. Chen et al. conducted a study demonstrating that silent information regulator 1 (SIRT1) levels can predict POF early in the disease course. They further found that combining serum SIRT1 with BISAP enhances the accuracy of mortality prediction [55].

Langmead et al. determined that a 5-cytokine panel (angiopoietin 2, hepatocyte growth factor, IL-8, resistin, and soluble tumor necrosis factor receptor 1A) accurately predicts POF early in the disease process, outperforming laboratory tests and clinical scores [56].

In addition to molecular biomarkers, simple hematologic ratios derived from routine tests have been evaluated for their predictive accuracy. In a study by Lu et al. on 446 patients between 2016 and 2019, it was found that high NLR levels were associated with a higher incidence of POF and may serve as an early predictor in individuals with hypertriglyceridemic pancreatitis (HTGP) [57].

In a retrospective study between 2018 and 2020 on 248 patients, Zhao et al. revealed that the CRP/serum albumin ratio is a promising, noninvasive prognostic indicator for patients with AP, capable of predicting SAP, mortality, pancreatic necrosis, and organ failure [58].

Lastly, Wang et al. determined that the red cell distribution width to serum albumin ratio (RDW-to-ALB ratio) is a promising early predictive marker for severe disease, complications, and mortality [59].

A summary of these biomarkers and their predictive implications is presented in Table 1.

**Table 1:** Summary of novel biomarkers for the prediction of organ failure in AP Ang-2, angiopoietin-2; AP, acute pancreatitis; BISAP, bedside index for severity in acute pancreatitis; CRP, C-reactive protein; CRP/ALB, C-reactive protein to albumin ratio; HGF, hepatocyte growth factor; HTGP, hypertriglyceridemic pancreatitis; MCTSI, modified computed tomography severity index; NLR, neutrophil-to-lymphocyte ratio; POF, persistent organ failure; RDW, red cell distribution width; RDW-to-ALB, red cell distribution width to albumin ratio; SAP, severe acute pancreatitis; SIRT1, silent information regulator 1; TNF-R1A, tumor necrosis factor receptor 1A

Authors	Study design	Markers	Number of studies/cases	Conclusion
Brown et al. [45]	Retrospective study	Hemoconcentration	128 patients	Patients lacking hemoconcentration seldom had pancreatic necrosis or organ failure.
Liu et al. [51]	Prospective study	Histones	236 patients	Within 48 hours of abdominal discomfort, quantitative evaluation of plasma histones can predict POF and mortality in AP patients.
Li et al. [52]	Retrospective study	Albumin	158 patients	In AP, serum albumin predicted organ failure.
Shu et al. [53]	Retrospective study	Lactate	329 patients	In individuals with SAP, initially increased arterial lactate is independently related to poor outcomes and mortality.
Zhang et al. [54]	Prospective study	Serum Ang-2	120 patients	High serum Ang-2 levels on admission may indicate POF in AP.
Chen et al. [55]	Prospective study	SIRT1	113 patients	High serum SIRT1 levels may indicate POF early. Combining serum SIRT1 and BISAP improved outcome prediction.
Langmead et al. [56]	Prospective study	5-cytokine panel (Ang-2, hepatocyte growth factor, IL-8, resistin, and soluble tumor necrosis factor receptor 1A)	60 patients	A 5-cytokine panel predicts POF early in the disease process better than laboratory tests and clinical scores.
Lu et al. [57]	Retrospective study	NLR	446 patients	Increased NLR was significantly associated with POF risk and may be an early independent predictor in HTGP patients.
Zhao et al. [58]	Retrospective study	C-reactive protein/albumin ratio	248 patients	CRP/ALB ratio may be a novel yet promising prognostic score in AP patients to predict SAP, mortality, pancreatic necrosis, and organ failure.
Wang et al. [59]	Retrospective study	Red cell distribution width to albumin ratio (RDW-to-ALB ratio)	301 patients	The RDW-to-ALB ratio predicts SAP as well as Ranson, BISAP, and MCTSI scores.

AI in organ failure in AP

The current developments in AI and machine learning (ML) demonstrate strong potential to forecast the severity and complications and treatment results of AP. The traditional Ranson, BISAP, and APACHE II models offer essential clinical guidance, yet their predictive power remains restricted by fixed variables, and their performance weakens during the initial stages of disease.

Given these limitations, AI-driven approaches have emerged as powerful tools for early and dynamic risk assessment in AP. AI models surpass traditional systems by processing multiple data types from clinical, biochemical, and radiological sources to enhance both predictive precision and immediate clinical choices. Recent models using gradient boosting frameworks in XGBoost, random forests, and support vector machines (SVMs) have led to significant improvements in predictive accuracy compared with traditional clinical scores.

In a single-center study of 441 patients, Zhou et al. developed an XGBoost-based model that identified severe cases with an AUC of 0.906 and accuracy of 0.902, outperforming other ML methods while highlighting CT severity index (CTSI), albumin, lactate dehydrogenase (LDH), and neutrophil count as key predictive factors [60]. Similarly, in a 648-patient cohort, Hong et al. created an interpretable random-forest model that achieved an AUC of 0.89 during cross-validation and 0.96 in the test set, substantially better than logistic regression or BISAP scoring [61].

Drawing on a larger, multicenter dataset of 5,460 admissions, Yuan et al. trained several ML algorithms and selected XGBoost as the best-performing approach for their APCU model, which reached AUC values of 0.95 (internal) and 0.873 (external) within the first 48 hours of presentation [62]. In another study, Yin et al. introduced the PrismSAP AI model, which combines CT-based radiomics with standard clinical features to achieve an external AUC of 0.916, surpassing Ranson, BISAP, and MCTSI [63].

Models and Input Features Evaluated in Recent Studies

Across these studies, investigators compared a range of supervised models beyond gradient boosting, including random forests and SVMs, with logistic regression typically used as a baseline comparator [60-62]. In multimodal pipelines that incorporate imaging, deep learning architectures have been applied to extract radiomic signatures from contrast-enhanced CT scans alongside structured clinical variables [63].

The most influential data features in these models frequently included routine laboratory markers (albumin, BUN, LDH, hematocrit, inflammatory indices, and neutrophil count) and radiologic indicators such as the CTSI or texture-based radiomic parameters reflecting necrosis burden, glandular edema, and peripancreatic inflammation. These predictors align closely with the underlying pathophysiology of early microcirculatory failure and systemic inflammation, explaining why ML models achieve superior discrimination during the first 24-48 hours of hospital admission.

AI systems maintain the ability to learn from new clinical information, allowing them to generate dynamic and individualized risk predictions. Radiomics-based models further enhance diagnostic accuracy by identifying early necrosis and distinguishing pancreatitis from other abdominal pathologies. Despite this progress, important barriers remain, including limited interpretability, inconsistent validation across diverse populations, data privacy concerns, and the need for user-friendly clinical interfaces.

Although several emerging biomarkers and AI-driven models show encouraging early performance, they are not yet ready to replace established clinical scores or routine bedside laboratory parameters. Most studies remain single-center and retrospective and lack standardized thresholds or external validation. At present, these tools should be viewed as complementary and hypothesis-generating rather than definitive clinical instruments. Larger, multicenter prospective studies are required before integrating these systems into standard practice.

Limitations 

The review article offers valuable insights but falls short in addressing a few limitations regarding its methodology, sample size, and definitions of organ failure, which diminishes comparability. The existing biomarker research relies primarily on retrospective and single-center data collection, while there is a noticeable absence of prospective multicenter validation, and many markers do not have established cutoff values or consistent assay protocols. The clinical application of these biomarkers is limited because they necessitate specialized equipment and costly testing, rendering them impractical for routine medical practice, especially in developing nations. Future research must focus on conducting comprehensive multicenter prospective trials that standardize assay methods while also integrating biomarkers into established risk assessment tools such as BISAP and APACHE II. The creation of affordable point-of-care diagnostic tools, in conjunction with AI systems, will enable clinicians to perform early patient assessments and develop personalized prediction models that combine biomarkers with clinical data. Research efforts should include studies that compare biomarkers against one another and evaluate long-term outcomes, including quality of life and costs, while examining diverse patient populations from developing regions to facilitate global clinical application.

## Conclusions

Early identification of patients with SAP remains fundamental to improving treatment outcomes. The detection of disease progression during critical early time periods remains challenging because traditional clinical scores and laboratory parameters prove insufficient. The pathophysiological mechanisms leading to organ failure can be detected through biomarkers such as ILs, angiopoietin-2, circulating histones, arterial lactate, and serum albumin. The combination of biomarkers with SIRS duration, BUN changes, and hemoconcentration can help healthcare providers to make better decisions about treatment timing. The predictive power of AI-based models that unite clinical data with laboratory results and imaging information becomes more accurate but faces barriers from inconsistent data formats and insufficient external testing. Research needs to create standardized biomarker testing methods while performing multicenter validation studies and developing affordable point-of-care diagnostic solutions. Integrating clinical tools with biochemical markers and AI systems through collaborative frameworks will create new approaches for early patient assessment and treatment protocols. The most effective approach for predicting organ failure in AP involves using a combination of established bedside assessment methods with new biomarkers and AI-based analytical systems to achieve better patient outcomes and resource management.
